# Determination of Anti-Phospholipase A2 and Anti-Thrombospondin Type 1 Domain-Containing Protein 7A in Latin Patients with Membranous Nephropathy

**DOI:** 10.3390/diagnostics13010017

**Published:** 2022-12-21

**Authors:** Ligia C. Battaini, Otavio T. Ranzani, Lia J. Marçal, Leila Antonangelo, Lecticia B. Jorge, Cristiane D. Bitencourt, Victoria Woronik, Denise M. A. Malheiros, Luis Yu

**Affiliations:** 1 Nephrology Division, University of Sao Paulo, Sao Paulo 04458-020, Brazil; 2 Barcelona Institute for Global Health, IS Global, 08036 Barcelona, Spain; 3 Pulmonary Division, University of Sao Paulo, Sao Paulo 04458-020, Brazil; 4 Pathology Department, University of Sao Paulo, Sao Paulo 04458-020, Brazil

**Keywords:** membranous nephropathy, antigens, antibodies, type M receptor of phospholipase A2 (PLA2R), thrombospondin type-1 domain-containing 7 A (THSD7A), nephrotic syndrome, proteinuria, autoimmune diseases, prevalence

## Abstract

Primary membranous nephropathy (MN) is caused by antibodies against podocyte antigens, especially the type M receptor of phospholipase A2 (PLA2R) and thrombospondin type-1 domain containing 7 A (THSD7A). This study’s aim was the determination of anti-PLA2R, anti-THSD7A serum antibodies, and anti-PLA2R renal tissue staining prevalence in a Latin population with MN, as well as evaluating their role as biomarkers for disease activity. The performance of the two anti-PLA2R serum diagnostic methods—ELISA and indirect immunofluorescence (IFI)—was evaluated for the diagnosis of MN. Fifty-nine patients, including 29 with MN, 18 with lupus membranous nephropathy (LMN) and 12 with focal and segmental glomerulosclerosis (FSGS), were evaluated for serum antibodies. Renal biopsies were also evaluated for the presence of anti-PLA2R staining. Twenty-one patients with MN were followed for 1 year. Patients with LMN and FSGS were negative for both antibodies. All 29 MN patients were negative for anti-THSD7A; 16 MN patients were positive for anti-PLA2R by ELISA and/or IFI, and 3 MN patients were positive for anti-PLA2R only by IFI. Thus, the anti-PLA2R ELISA test demonstrated 45% sensitivity and 97% specificity, while the IFI test showed, respectively, 55% and 100% in our MN patients. Among the 28 MN renal biopsies, 20 presented anti-PLA2R positive staining, corresponding to a 72% sensitivity. Positive correlations were observed between the anti-PLA2R ELISA titer and proteinuria. In conclusion, determination of anti-PLA2R antibodies in the MN Latin population showed similar rates to those reported for other populations. The anti-PLA2R serum levels correlated with MN disease activity.

## 1. Introduction

Membranous nephropathy (MN) is a common cause of nephrotic syndrome in adults [1] and considered the second most common cause of glomerulopathies in Brazil [2,3]. Different incidences may reflect country specificities, different biopsy indications or socioeconomic, ethnic and environmental differences in diverse populations [4].

The pathogenesis of this nephropathy has not been determined since Jones’ initial description [5] in 1957. Experimental studies performed by Heymann et al. in 1959, discovered that the active immunization of rats with renal extracts induced kidney disease similar to human MN [6]. This experimental model, Heymann’s nephritis, has long been used as an autoimmune disease model with autoantibody production. However, no definite connection could be established between this experimental model and human nephropathy [7]. In 2002, Ronco et al. firstly demonstrated the development of MN in newborns of mothers deficient of neutral endopeptidase, an endogenous protein associated with podocytes, against which mothers’ antibodies were directed, resulting in immune complex formation in the newborns’ kidneys and nephrotic syndrome [8,9,10].

In 2009, the seminal discovery of antibodies against receptors located in podocytes of normal and MN human kidneys was reported [11]. Beck et al. described the role of anti-phospholipase A2 receptor (anti-PLA2R) autoantibodies in the pathogenesis of a substantial fraction (70–80%) of patients with primary MN. Two years later, Debiec and Ronco described the relationship between the presence of the serum antibody and glomerular deposits of this antibody in a French cohort. The sensitivity of anti-PLA2R tests performed on serum and renal tissue was 57% and 74%, respectively [12]. These data demonstrated the complementarity of the diagnostic tests (serum and tissue) and their good sensitivity and high specificity for MN diagnosis [12,13,14].

In 2014, Tomas et al. identified another antibody in a population of negative anti-PLA2R MN: anti-thrombospondin type-1 domain-containing 7A (THSD7A), which has a prevalence between 3 and 5% in the studied populations [15]. Recently, Sethi et al., using laser microdissection of the glomeruli and mass spectrometry, have identified new antigens in the glomeruli of patients with MN negative for anti-PLA2R and anti-THSD7A, such as exostosin 1 and 2 in the glomeruli of patients with MN secondary to autoimmune diseases, especially SLE; NELL-1 (neural epidermal growth factor-like 1 protein) in a cohort of MN patients [16,17]; semaphorin 3B (Sema 3B), another uncommon antigen that is more prevalent in the pediatric population; and protocadherin 7 (PCDH7), whose role in MN is yet to be determined. Further studies are needed to confirm the prevalence of these new proteins and confirm their role as antigens in MN [18].

Among the available assays for the detection of anti-PLA2R antibodies, the Western blot (WB) technique, which has been used in pioneering studies, is, however, hardly applicable in clinical practice. The first commercially available assay was the anti-PLA2R indirect immunofluorescence test (CBA—IFI, Euroimunn, Luebeck, Germany), although more sensitive, it does not allow quantification, rendering it less useful for monitoring disease progression and therapeutic response. Subsequently, an anti-PLA2R ELISA test (Euroimunn, Luebeck, Germany) was developed, and it has been mostly employed in clinical practice because of the quantitative determination assay.

Proteinuria and renal function monitoring have been used in clinical practice to assess disease progression and therapeutic indications. However, since anti-PLA2R antibody levels are considered highly sensitive and specific for the diagnosis of patients with primary MN, and serum level sequential determinations may allow for disease activity monitoring and therapeutic response, the use of anti-PLA2R serum levels as a biomarker capable of reflecting the disease’s immunological activity and indicating clinical course can substantially improve patient care.

Therefore, the objectives of this study were to determine anti-PLA2R, anti-THSD7A serum antibodies and anti-PLA2R renal tissue staining prevalence in a Latin population with primary MN, and to evaluate their role as biomarkers for disease activity. Additionally, the performance of the two available anti-PLA2R serum diagnostic methods—ELISA and IFI— was evaluated for the diagnosis of MN.

## 2. Materials and Methods

### 2.1. Studied Population

The presence of serum antibodies (anti-PLA2R and anti-THSD7A) was evaluated in 59 patients from the Glomerulopathies Outpatient Clinic at Hospital das Clinicas, University of São Paulo, Brazil (HCFMUSP). Twenty-nine out of these patients were diagnosed with MN, 18 were diagnosed with lupus membranous nephropathy (LMN) and 12 were diagnosed with focal and segmental glomerulosclerosis (FSGS), with the latter two groups included as the control group. All patients had a histological diagnosis confirmed by renal biopsy. The MN patients included in the study were investigated for secondary causes of the disease. Patients who had been administered drugs or medications capable of causing MN were excluded. The following laboratory tests were recorded: antinuclear antibody (ANA); anti-DNA; serum complement; serologies for hepatitis B and C, HIV and syphilis and investigation of neoplasia according to age and symptoms. Renal biopsies were analyzed for the presence of anti-PLA2R antibody in the renal tissue of 28 patients with histological diagnosis of MN, 17 with diagnosis of LMN and 7 with diagnosis of FSGS.

The study protocol was approved by the Research Ethics Committee of HCFMUSP, and patients signed a consent form.

### 2.2. Data Collection

The following demographic data were collected from medical records: age, sex, ethnicity, clinical history, personal and family history and drug treatment, including the use of anti-proteinuric medications and immunosuppressants (cyclosporine, cyclophosphamide, tacrolimus, corticosteroids, and rituximab). In addition, the following laboratory data were collected: urea, creatinine and electrolytes, urine analysis and 24 h proteinuria, biochemical and hematological parameters (blood count, protein electrophoresis, cholesterol, triglycerides, glycemia, glycosylated hemoglobin, and C-reactive protein), serologies (HIV, hepatitis B and C) and immunological parameters (SLE, serum complement and fractions).

### 2.3. Determination of Serum Autoantibodies

Blood samples were collected from all patients at the time of inclusion in the study for the measurement of anti-PLA2R and anti-THSD7A levels. Anti-PLA2R was determined using two commercially available methods, anti-PLA2R ELISA—from Euroimmunn AG (Lubeck, Germany-code EA 1254-9601 G)—and anti-PLA2R Indirect Immunofluorescence (IFI)—from Euroimmunn AG (Lubeck, Germany—CA code 1254-0101). The determination of anti-THSD7A antibodies was carried out using the commercial kit anti-thrombospondin type-1 domain-containing protein 7A—Euroimmunn AG (Lubeck, Germany—code FA 1254-1005-51) by indirect immunofluorescence. For the anti-PLA2R ELISA test, a value greater than 20 RU/mL was considered positive, between 14–20 RU/mL was considered indeterminate and <14 RU/mL was considered negative. Two independent observers evaluated all renal tissue slides using a microscope for immunofluorescence reading.

### 2.4. Determination of Anti-PLA2R in Renal Tissue

Renal tissue obtained from paraffin blocks was stained for anti-PLA2R by immunohistochemistry using monoclonal anti-anti-PLA2R antibodies produced in mice by Prestige Antibodies and Atlas Antibodies (Sigma-Aldrich/Merck, code AMAB 90772, St. Louis, MO, USA).

### 2.5. Follow-Up

Twenty-one patients with primary MN were followed, and their serum anti-PLA2R and anti-THSD7A antibodies were measured at 6 and 12 months of follow-up from the time of inclusion in the study. Laboratory data, such as renal function, serum albumin and proteinuria levels were also analyzed, and clinical signs of disease activity and medications were recorded.

### 2.6. Statistical Analysis

A sample size of 28 cases and 28 controls was estimated based on a diagnostic case–control design, considering an anti-PLA2R sensitivity of 75%, with delta of 25% (i.e., lower limit of 50%), alpha of 5% and statistical power of 80%. A value of 86% power and an expected 90% specificity, 5% alpha and 20% delta were guaranteed for the 28 cases and controls, and the Flahault method was utilized.

Frequencies and proportions for categorical variables and the mean ± SD or median [p25–p75] for continuous variables were determined as appropriate. Comparisons between two groups were performed with Student’s T or Mann–Whitney tests, as well as ANOVA, and Kruskal–Wallis tests were used for comparisons among three or more groups. Simple linear correlations were estimated by Pearson’s or Spearman’s correlation coefficients (Spearman’s rho) as appropriate. A normal distribution was evaluated based on a visual inspection of the histogram.

Sensitivity, specificity, accuracy, positive and negative likelihood ratios, and diagnostic odds ratios were calculated using standard formulas. The positive and negative predictive values were not estimated because of the case–control design. The area under the ROC curve was calculated to evaluate the discrimination performance of the anti-PLA2R titers.

A linear mixed model with a random intercept for patient and time to account for the longitudinal and repeated data structure was calculated. All analyses were performed using the R program (R Foundation for Statistical Computing, Vienna, Austria), version 4.0.2.

## 3. Results

MN patients presented a mean age of 46 (±14) years and were predominantly male. Thirteen patients (45%) were hypertensive (SAH), and three patients (10%) were diagnosed with diabetes mellitus (DM). The time elapsed between renal biopsy and inclusion in the study was shorter in MN patients than in the control group, with a median of 17 (5–32) months in the case group vs. 38 (22–49) months in the control group (*p* < 0.001).

At the time of biopsy, groups did not differ regarding the serum creatinine level, leukocyte count or red blood cell count in the urine. MN patients had lower levels of serum albumin than the control group [1.6 (1.2–2.0) g/dL vs. 2.6 (2.0–3.4) g/dL, *p* < 0.001] and higher values of proteinuria [6.9 (3.9–9.3) g/24 h vs. 2.8 (1.4–5.4) g/24 h, *p* = 0.003] (Table 1). 

When assessing data at the time of inclusion in the study (serum antibody determination) compared with the control group, patients with MN had higher values of serum creatinine: 1.20 (0.98–1.64) mg/dL vs. 0.79 (0.61–1.22) mg/dL, lower levels of serum albumin—2.9 (2.7–3.8) g/dL vs. 3.9 (3.3–4.2) g/dL, and higher levels of proteinuria—5.3 (0.9–8.4) g/24 h vs. 0.4 (0.1–1.1) g/24 h (cases vs. control, respectively) (Table 2).

Among patients with MN, 15 patients (52%) were using ACE inhibitors and/or ARBs at the time of biopsy and 26 patients (90%) were using ACE inhibitors and/or ARBs at the time of inclusion in the study. Approximately 15 patients (52%) were using immunosuppressants at the time of inclusion, and 26 patients (90%) were using or had received immunosuppression since the time of the biopsy.

### 3.1. Prevalence of Autoantibodies

All control patients, including 18 patients with LMN and 12 patients with FSGS, were negative for anti-PLA2R and anti-THSD7A antibodies except for one patient with FSGS who was positive for anti-PLA2R (positive by ELISA, negative by IFI). In addition, the 29 MN patients were negative for anti-THSD7A.

Among the 29 MN patients, a positive anti-PLA2R ELISA test was found in 13 patients (45%), while a positive anti-PLA2R IFI test was found in 16 patients (55%) (Table 3). Differences were not observed regarding age, gender, or comorbidities (SAH and DM) between anti-PLA2R positive and negative MN patients. The time elapsed between the time of biopsy and the determination of antibodies was longer in anti-PLA2R-negative patients, with a median of 29 months (25–35) vs. 6 months (2–14) in anti-PLA2R-positive patients (*p* = 0.004).

At the time of renal biopsy, the two groups were similar regarding serum creatinine, serum albumin, proteinuria, and urine sediment. However, at the time of inclusion in the study, positive anti-PLA2R patients presented significantly lower values of serum albumin: 2.9 (1.7–2.9) g/dL vs. 3.2 (2.9–3.9) g/dL in the controls (*p* = 0.033), and higher proteinuria values: 8.3 (2.1–8.5) g/24 h vs. 1.6 (0.4–5.1) g/24 h in the controls (*p* = 0.003). In addition, they presented altered urine sediment, with higher values of leukocyturia and hematuria (Table 4).

### 3.2. Comparison between Anti-PLA2R Methods

At the end of the study, the anti-PLA2R ELISA test showed a sensitivity of 45%, while the anti-PLA2R IFI test showed a sensitivity of 55%. The anti-PLA2R ELISA test had a specificity of 97%, while the IFI was 100%.

When assessing patients with a timeframe between biopsy and antibody determination of less than 1 year (20 patients, including 14 patients with MN), the anti-PLA2R diagnosis sensitivity by ELISA and/or IFI increased to 79% and maintained 100% specificity. At the time of inclusion in the study, these 14 patients with MN presented significantly greater proteinuria than those with a biopsy timeframe greater than 1 year [8.4 (5.7–8.5) g/24 h vs. 1.6 (0.6–2.7) g/24h, respectively, *p* < 0.001], they also presented lower serum albumin [1.9 (1.6–2.9) g/dL vs. 3.8 (2.9–4.1) g/dL, *p* < 0.002] and greater hematuria [6 (2–14) vs. 2 (0–4) red blood cells/field, *p* = 0.052].

Those four patients who tested positive with the anti-PLA2R IFI test and negative with the ELISA test had the following titers in the latter test: 15.75, 17.37, 19.18 and 19.79 RU/mL, which would be positive if a cutoff value of 14 RU/mL for ELISA was considered as proposed by other investigators [19].

**Figure 1:**
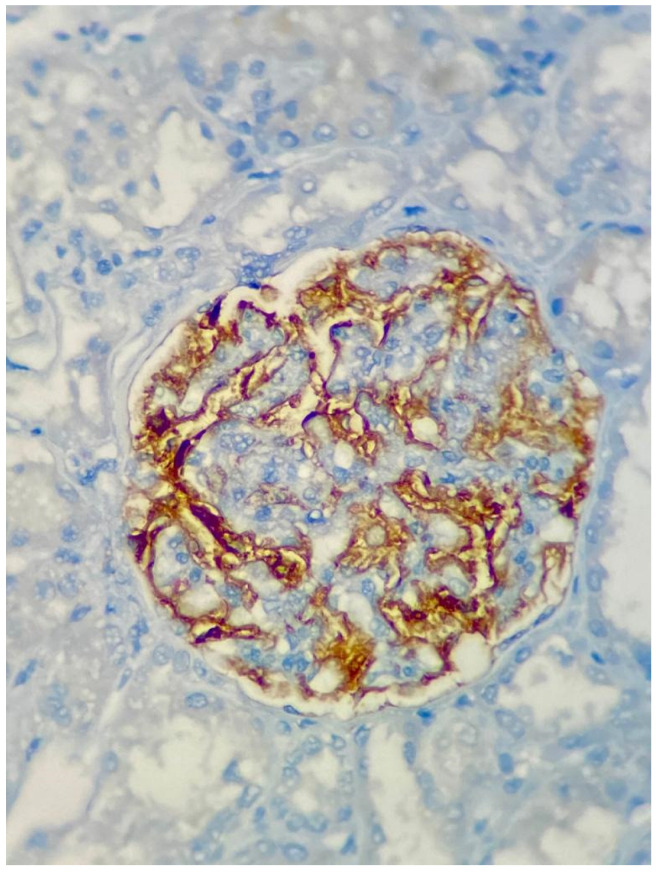
Representative immunohistochemistry for a positive anti-PLA2R in MN kidney biopsy.

A total of 52 kidney biopsy slides were evaluated, including 28 patients with histological diagnosis of MN, 17 patients with LMN and seven patients with FSGS. The slides were divided according to the degree of tissue staining: light was considered negative and moderate and strong staining were considered positive, as illustrated in Figure 1.

Among the seven patients with FSGS, five had serum samples, which were all negative for autoantibodies. In the renal tissue, four presented negative anti-PLA2R staining and three presented positive anti-PLA2R staining. Among all 17 patients with LMN with negative serum samples for autoantibodies, only two showed positive tissue staining.

A total of 28 patients with primary MN were analyzed: 20 out of 28 patients presented positive anti-PLA2R staining on renal biopsy while eight patients were negative, resulting in a sensitivity of 72%. Among all 28 MN patients, 23 patients had positive serum samples for anti-PLA2R, with seven patients showing negative results in both renal tissue and serum and 16 patients showing anti-PLA2R-positive results in the tissue. Thirteen out of 16 anti-PLA2R tissue-positive patients were also serum positive, while three patients were serum-negative, resulting in a sensitivity of 70% in the tissue and 57% in the blood. None of the patients that were positive for antibodies in the serum showed negative biopsy results.

Serum measurements were not performed simultaneously with renal biopsy, and an interval of years (average of 19 months) occasionally occurred, which precluded the interpretation of the results and prevented comparisons between the sensitivities of the serological test and tissue staining. Among the 21 patients that were followed for 12 months, 20 of them were also evaluated for anti-PLA2R on renal biopsy. A comparison of patients with positive (N = 14) vs. negative (N = 6) anti-PLA2R biopsy staining at the time of inclusion demonstrated that patients with a positive biopsy presented higher PTU: 4.2 (1.7–8.5) g/24 h vs. 0.3 (0.2–5.5) g/24 h (*p* = 0.031) and lower serum albumin: 2.7 (1.7–3.8) g/dL vs. 3.9 (2.9–4.2) g/dL (*p* = 0.037), respectively, and similar results were observed when comparing patients with positive vs. negative serum anti-PLA2R antibodies.

### 3.3. Correlation between Anti-PLA2R Titers and Disease Activity

A total of 21 patients with MN had another blood sample collected 6 and 12 months after follow-up. After 6 months, two patients changed the autoantibody profile. One patient that was anti-PLA2R positive by the ELISA and IFI tests at the time of inclusion became negative by both tests, which was accompanied by a drop in proteinuria from 9.0 g/24 h to 2.31 g/24 h. This patient was on immunosuppressants. A second patient who initially presented a negative anti-PLA2R ELISA test (19 RU/mL) and a positive IFI test, became anti-PLA2R-positive by the ELISA test after 6 months, with a titer of 28 RU/mL, and his proteinuria increased from 0.69 g/24 h to greater than 6.0 g/24 h. This patient was using immunosuppressants at the beginning of the study, but his medication was suspended before the second blood collection.

After 1 year of inclusion, one patient who presented an anti-PLA2R-positive test by ELISA and IFI over the course of the follow-up showed a decrease in anti-PLA2R ELISA titers (1410 > 1760 > 15.89 RU/mL) but remained anti-PLA2R-positive by IFI, which was not accompanied by proteinuria reduction. This patient had not tolerated immunosuppression with cyclophosphamide and received rituximab 2 months before the first collection. Another patient who had anti-PLA2R-negative test by ELISA and IFI over the course of the follow-up became anti-PLA2R positive at the third collection, which was preceded by the suspension of immunosuppression (cyclosporine) and presented a concomitant increase in proteinuria (6.0 g/24 h > 0.3 g/24 h > 6.42 g/24 h).

At the time of inclusion in the study, the median anti-PLA2R ELISA titer among the 29 MN patients was 15.7 (1.21–179) RU/mL. Among the 21 patients followed for a period of 12 months, a reduction in these titers over time was observed, with a median of 5.55 (0.87–96.0) RU/mL at 6 months. After 12 months, a median of 5.24 (0.26–47.1) RU/mL was detected. This variation in antibody titers was accompanied by a reduction in proteinuria levels and an increase in serum albumin. The median proteinuria at the initial moment was 5.27 (1.0–8.4) g/24 h; after 6 months, the median proteinuria was 1.54 (0.3–8.5) g/24 h; and after 12 months, the median proteinuria was 1.55 (0.2–4.9) g/24 h. The serum albumin levels at inclusion, 6 months and 12 months were: 2.9 (2.7–3.8) > 4.3 (3.2–4.6) > 3.9 (3.6–4.6) g/dL, respectively.

When correlating the anti-PLA2R ELISA titer with 24 h proteinuria at the time of inclusion and throughout the follow-up, a positive correlation was observed (Spearman’s rho: 0.554 at inclusion, 0.696 at 6 months and 0.709 at 12 months; *p* = 0.001); that is, higher proteinuria corresponded to higher anti-PLA2R ELISA titers. Regarding serum albumin, an inverse relationship was observed, with a lower serum albumin level corresponding to higher anti-PLA2R ELISA titers (Spearman’s rho: −0.645 at inclusion, −0.724 at 6 months and −0.742 at 12 months; *p* = 0.001). There was no correlation between serum creatinine levels and anti-PLA2R ELISA titers.

The pattern described above was maintained throughout the follow-up period. In the linear model adjusted for repeated antibody measurement during the three collection periods, the correlation coefficient between anti-PLA2R and PTU titers was 0.226 (*p* < 0.001) and between anti-PLA2R titers and serum albumin it was −0.057 (*p* <0.001). In the model adjusted for age, sex, SAH and DM, the correlation coefficient between anti-PLA2R titers and PTU was 0.188 (*p* = 0.001) and between anti-PLA2R titers and albumin it was −0.052 (*p* < 0.001).

When assessing the remission rate among the 21 patients followed up for 12 months, a greater probability of partial or total remission of PTU was observed in patients with negative vs. positive anti-PLA2R test (89% vs. 33%, *p* = 0.024). There was no relationship between antibody positivity and the progression of CKD. Twenty out of 21 patients had their histology evaluated for the presence of anti-PLA2R staining. Patients with anti-PLA2R-negative staining in the renal biopsy were more likely to achieve partial or complete remission; however, there was no significant difference.

## 4. Discussion

Determination of anti-PLA2R antibodies in the MN Latin population showed similar rates as reported for other populations. The anti-PLA2R serum levels correlated with MN disease activity. 

All included patients, regardless of the underlying disease, were negative for anti-THSD7A antibodies. Data from the literature show that no healthy individual or with proteinuric diseases other than MN tested positive for anti-THSD7A despite its specificity being close to 100% [15,20]. However, the sensitivity was low, ranging from 3% in Europe and the United States to 9% in Japan [15,20,21,22]. Considering the present sample size and the method sensitivity, the absence of positive results was expected.

On the other hand, anti-PLA2R antibody determinations were performed using two available techniques, ELISA and IFI tests. As described by different authors, all patients diagnosed with LMN and FSGS (control group) were negative according to the IFI technique, which provided 100% specificity in this group. When evaluated by anti-PLA2R ELISA test, the specificity in this group was 97%. Only one patient with a clinical and histological diagnosis compatible with FSGS had a low titer of anti-PLA2R (23 RU/mL) result by ELISA and a negative anti-PLA2R IFI test, which may be considered a false positive [23].

Among our MN patients, 55% were positive according to either one of the techniques employed, and similar data were found in other cohorts, where 50–80% of MN patients tested positive for anti-PLA2R antibodies (sensitivity), with a specificity ranging from 90–97% [23,24,25].

In this sample, patients with an interval of less than 12 months between biopsy and antibody collection compared to those with an interval greater than 1 year had greater anti-PLA2R serum positivity (79%) and presented greater proteinuria [8.4 (5.7–8.5) vs. 1.6 (0.6–2.7) g/24 h, *p* = 0.001] and lower serum albumin [1.9 (1.6–2.9) vs. 3.7 (2.9–3.9) g/dL, *p* = 0.002], indicating disease activity in the former group.

Newly diagnosed populations presented positivity between 60.2% and 80%, which was higher than that found in prevalent populations [19,26,27,28]. Different reasons may contribute for this variability among studies and explain the greater positivity when the interval between biopsy and antibody collection is restricted to less than 12 months. The type of test used (WB, IFI and ELISA), ethnic differences and the timing of antibody collection in relation to the course of the disease are some of the main reasons, especially the latter, that appear to be most relevant. Moreover, patients with a shorter interval between biopsy and antibody collection are more likely to have active disease.

Studies in different populations demonstrated an association between disease activity, mainly represented by proteinuria and the presence of antibodies. Anti-PLA2R titers often mirror disease activity, so when antibody titers are high, proteinuria usually is also high. It is well established that antibody levels fluctuate significantly during the course of the disease [29].

Dai et al. performed a meta-analysis and showed greater accuracy of anti-PLA2R discrimination according to the degree of proteinuria [23]. In a subgroup analysis, the anti-PLA2R test showed greater accuracy among patients with nephrotic syndrome (AUC 0.83) than those without nephrotic proteinuria (AUC 0.47). This difference could be justified by the antibody level fluctuation according to the disease activity due to a spontaneous decrease of serum level (remission) or because of the use of immunosuppression [27,30,31]. In our study, patients with positive anti-PLA2R titers had higher proteinuria than negative patients [8.3 (2.1–8.5) g/24 h vs. 1.6 (0.4–5.1) g/24 h *p* = 0.003]. 

There is a correlation between antibody positivity and proteinuria but also between antibody titers and proteinuria values. According to data obtained at the time of inclusion, a positive correlation was found between anti-PLA2R titers by ELISA and 24 h proteinuria (Spearman rho: 0.554, *p* = 0.001); that is, the greater anti-PLA2R titers, the greater the proteinuria. This observation has been demonstrated by different authors in different populations [27,32,33,34]. 

In this study, anti-PLA2R IFI and ELISA techniques were compared regarding sensitivity and specificity, and the results varied according to the moment of determination.

At the end of the study, a sensitivity of 45% and specificity of 97% were obtained for anti-PLA2R ELISA test and a sensitivity of 55% and specificity of 100% were obtained for anti-PLA2R IFI test, with an agreement of 86% and a correlation of 0.72. These findings are compatible with those described in the literature, which shows a good correlation between the ELISA and IFI tests despite the IFI test having greater sensitivity [24,29,35,36,37,38].

In 2014, Behnert et al. compared different techniques in a cohort of 157 MN patients. There was a good correlation between the techniques employed, with an agreement between the ELISA and the IFI tests of 85.9% and a correlation of 0.72, which was similar to our study [38].

Anti-PLA2R ELISA titers over 20 RU/mL are considered positive according to the manufacturer’s instructions. However, some authors have suggested that the reference value should be reduced to 2 RU/mL or 14 RU/mL. These authors based their suggestion on studies that showed increased sensitivity without a loss of specificity [37,38]. Considering the cutoff value of 14 RU/mL, the sensitivity of the anti-PLA2R ELISA test would be equivalent to that found for the IFI test at 55% of our population because all patients with a negative anti-PLA2R ELISA test and positive anti-PLA2R IFI test presented titers greater than 14 RU/ml in our sample.

Tissue staining for the anti-PLA2R antibody was first described in 2011, and since then it has been used to complement the diagnosis of patients with suspected MN. Different studies have compared the sensitivity of tissue staining with serum antibody measurements, and a greater sensitivity of tissue staining was observed. This anti-PLA2R tissue staining has also been used to differentiate primary causes from secondary causes of MN.

Fifty-two biopsies from patients with MN (N = 28), LMN (N = 17) and FSGS (N = 7) were retrospectively evaluated using the immunohistochemistry technique, which was first used for this purpose in our institution. As described in the literature, biopsy of normal kidneys shows weak positivity for anti-PLA2R, and this degree of staining is considered negative [14,39].

Among the biopsies evaluated, 2/17 patients diagnosed with LMN and 3/7 diagnosed with FSGS were positive. Studies have shown antibody positivity for secondary causes of MN, which are mainly related to HBV, HCV and sarcoidosis and rarely described in patients with SLE or FSGS [14,39,40,41].

In addition, among the 28 patients with primary MN, 20 patients (72%) showed a positive anti-PLA2R result in the renal tissue, demonstrating that in the absence of serum antibody measurements, tissue staining may play an important role in the diagnosis of primary MN. Furthermore, tissue staining may allow for a retrospective diagnosis, which may be important for the evaluation of patients who are candidates for kidney transplantation.

Renal biopsy staining and serum data were obtained for 23 MN patients. Seven out of 23 patients were both anti-PLA2R negative for blood and tissue; 16 patients were positive for anti-PLA2R antibodies on renal biopsy, among them three had negative serology and 13 presented serum anti-PLA2R positivity. No patients with positive serology and negative renal biopsy were found. It was not possible to establish a correlation between the sensitivity of these two methods (serum and tissue) because of the large interval between the biopsy and serum antibody determinations, which had a mean interval of 19 months. In this time interval, spontaneous or immunosuppression-induced remission may have occurred with serum antibody clearing. Only the concomitant determination of serum antibodies and renal biopsy staining would allow an adequate sensitivity comparison between these two methods.

A total of 29 MN patients were included in the study, and 21 patients were followed up for 12 months, with additional serum antibody determinations on 6 and 12 months and concomitant evaluations of PTU, serum albumin and serum creatinine. Throughout the follow-up, the positive relationship between the PTU and anti-PLA2R ELISA titers and the negative relationship with serum albumin were maintained. Four patients had changes in their initial serum autoantibody profile, which were accompanied by changes in proteinuria levels and preceded by the initiation or suspension of immunosuppressants.

When assessing the likelihood of remission according to the presence or absence of antibodies during follow-up, an inverse relationship was observed: patients who were positive for anti-PLA2R antibodies in both, serum and renal biopsy, showed a lower remission rate, which was defined as partial or incomplete proteinuria reduction. In fact, different studies have found a relationship between antibody titers and the likelihood of remission. Patients with lower anti-PLA2R ELISA titers evolved more favorably, while patients with higher titers presented a greater possibility of developing nephrotic syndrome and kidney function loss. However, among our patients, there was no correlation between anti-PLA2R-positive antibodies and progression to CKD [24].

Serial measurements of the antibodies titers provide even more accurate prognostic data. Decreasing antibody levels throughout the follow-up preceded proteinuria reduction. Serial measurements have also been used for prognostic determination and treatment indication or cessation in different centers across the world.

Finally, this study presents limitations, including the small sample size despite achieving the calculated sample; the unicenter nature of the study may not represent the entire population and outcomes were based on Cr values rather than the estimated glomerular filtration rate, a more accurate renal filtration measurement. Nevertheless, the prevalence of anti-PLA2R antibodies in a Latin MN population was found to be similar to other populations.

## 5. Conclusions

This study aimed at estimating the prevalence of anti-THSD7A and anti-PLA2R serum antibodies in patients with primary MN in a Latin population using both the IFI and ELISA techniques, as well as anti-PLA2R renal tissue staining by immunohistochemistry. All patients were negative for anti-THSD7A serum antibodies. Positivity for anti-PLA2R antibodies ranged from 55 to 79% depending on the time of the renal biopsy. Both anti-PLA2R diagnostic techniques showed a good correlation, with the IFI test showing greater sensitivity (55%), with both tests reaching a specificity close to 100%. When assessing the sensitivity of anti-PLA2R staining by immunohistochemistry in renal tissue, a 72% positivity was obtained in patients with primary NM. In addition, a positive correlation was obtained between the anti-PLA2R serum titers and proteinuria, reflecting MN disease activity.

## Figures and Tables

**Table 1:** Laboratory data at the time of renal biopsy.

	Cases (N = 29)	Controls (N = 30)	*p*
Serum creatinine (mg/dL)	0.99 [0.88–1.22]	1.10 [0.63–1.57]	0.81
Serum albumin (g/dL)	1.6 [1.2–2.0]	2.6 [2.0–3.4]	<0.001
Urine (unit/field)			
Leukocytes	3 [2–5]	4 [2–12]	0.198
Red cells	8 [2–15]	7 [3–72]	0.72
Proteinuria 24 h (g/24 h)	6.9 [3.9–9.3]	2.8 [1.4–5.4]	0.003
<3.5	6 (23%)	16 (53%)	
3.5–6.0	6 (23%)	10 (33%)	0.004
>6.0	14 (54%)	4 (13%)	

**Table 2:** Laboratory data in cases and control patients at the time of inclusion in the study.

	Cases (N = 29)	Controls (N = 30)	*p*
Serum creatinine (mg/dL)	1.20 [0.98–1.64]	0.79 [0.61–1.22]	0.001
Serum albumin (g/dL)	2.9 [2.7–3.8]	3.9 [3.3–4.2]	0.011
Urine (unit/field)			
Leukocytes	1 [0–4]	2 [0–11]	0.38
Red cells	2 [1–9]	1 [0–6]	0.170
Proteinuria 24 h (g/24 h)	5.3 [0.9–8.4]	0.4 [0.1–1.1]	<0.001
<3.5	13 (46%)	27 (93%)	
3.5–6.0	4 (14%)	1 (4%)	0.001
>6.0	11 (39%)	1 (4%)	

**Table 3:** Autoantibodies’ profile by anti-PLA2R ELISA and IFI tests in patients with primary MN.

	Sensitivity	Specificity
Anti-PLA_2_R ELISA >20 RU/ml	45% (13/29)	97%
Anti-PLA_2_R, Positive IFI	55% (16/29)	100%
Anti-PLA_2_R Positive ELISA and/or IFI	55%	97%

**Table 4:** Comparison between negative and positive anti-PLA2R serum MN patients at the time of inclusion in the study.

	MN Anti-PLA2R Negative (N = 13)	NM Anti-PLA2R Positive (N = 16)	*p*
Serum creatinine (mg/dL)	1.17 [1.00–1.75]	1.22 [0.96–1.53]	0.84
Serum albumin (g/dL)	3.5 [2.9–4.1]	2.9 [1.7–2.9]	0.018
Urine (unit/field)			
Leukocytes	0 [0–1]	3 [1–5]	0.012
Red cells	2 [0–2]	5 [2–12]	0.011
Proteinuria 24 h (g/24 h)	1.3 [0.3–3.9]	8.0 [3.9–8.5]	0.001
<3.5	9 (75%)	4 (25%)	
3.5–6.0	2 (17%)	2 (13%)	0.012
>6.0	1 (8%)	10 (62%)	

## Data Availability

Not applicable.
